# Construction and Validation of a Platinum Sensitivity Predictive Model With Multiple Genomic Variations for Epithelial Ovarian Cancer

**DOI:** 10.3389/fonc.2021.725264

**Published:** 2021-09-16

**Authors:** Hong Zheng, Tong Shu, Shan Zhu, Chao Zhang, Min Gao, Nan Zhang, Hongguo Wang, Jie Yuan, Zaixian Tai, Xuefeng Xia, Yuting Yi, Jin Li, Yanfang Guan, Yang Xiang, Yunong Gao

**Affiliations:** ^1^Department of Gynecologic Oncology, Peking University Cancer Hospital & Institute, Beijing, China; ^2^Department of Obstetrics and Gynecology, Peking Union Medical College Hospital, Chinese Academy of Medical Sciences and Peking Union Medical College, Beijing, China; ^3^Geneplus-Shenzhen, Shenzhen, China; ^4^Geneplus-Beijing, Beijing, China; ^5^Department of Computer Science and Technology, School of Electronic and Information Engineering, Xi’an Jiaotong University, Xi’an, China

**Keywords:** biomarker, next generation sequencing, ovarian cancer, platinum sensitivity, ROC curve

## Abstract

Platinum-based chemotherapy is still the standard of care after cytoreductive surgery in the first-line treatment for epithelial ovarian cancer. This study aims to integrate novel biomarkers for predicting platinum sensitivity in EOC after initial cytoreductive surgery precisely. To this end, 60 patients were recruited from September 2014 to October 2019. Based on the duration of progress-free survival, 44 and 16 patients were assigned to platinum-sensitive and platinum-resistant group, respectively. Next generation sequencing was performed to dissect the genomic features of ovarian tumors obtained from surgery. Multiple genomic variations were compared between two groups, including single-nucleotide variant, single base or indel signature, loss of heterozygosity (LOH), whole-genome duplication (WGD), and others. The results demonstrated that patients with characteristics including positive SBS10a signature (p < 0.05), or *FAM175A* LOH (p < 0.01), or negative WGD (p < 0.01) were significantly enriched in platinum-sensitive group. Consistently, patients with positive SBS10a signature (15.8 *vs.* 10.1 months, p < 0.05), or *FAM175A* LOH (16.5 *vs.* 9.2 months, p < 0.05), or negative WGD (16.5 *vs.* 9.1 months, p < 0.05) have significantly longer PFS than those without these genetic features. By integrating these three biomarkers, a lasso regression model was employed to train and test for all patients, with the AUC value 0.864 in platinum sensitivity prediction. Notably, 388 ovarian cancer patients from TCGA dataset were leveraged as independent validation cohort with AUC value 0.808, suggesting the favorable performance and reliability of this model.

## Introduction

Epithelial ovarian cancer (EOC) remains the most aggressive and lethal disease of all gynecologic malignancies. Seventy percent of the patients are in advanced stage at initial diagnosis. Although the majority of these patients respond to first-line treatment, around 70% of them recur within 2 years (1). Treatment strategy for recurrent EOC is mainly based on platinum sensitivity which is defined by the duration of progression free survival (PFS). PFS less than 6 months is considered platinum resistant, while PFS more than 6 months reveals platinum sensitive (2). However, there are few effective methods to predict the timepoint of recurrence and prejudge its platinum sensitivity. Now platinum-based chemotherapy is still the standard of care after cytoreductive surgery in the first-line treatment in EOC. The prediction of platinum sensitivity before chemotherapy could assist us applying more individualized regimen as well as other therapeutic modalities.

Treatment for EOC has begun to develop into individualized management following the new era of precise medicine. It was reflected in the appropriate systemic treatment based on clinical characteristics and intrinsic genomic alterations of patients. Due to increasing advances in sequencing technologies and decreasing costs, our understanding of molecular basis for ovarian cancer has improved extensively (3). Accumulating evidence have proved that the “gene testing” could provide significant impact on treatment options. Predominantly, tumor with *BRCA* mutations and homologous recombination deficiency (HRD), which emerged in 25% and 50% of high grade serous ovarian cancer respectively, were associated with better response to platinum and *PARP* inhibitors (4). In addition, the exploration of other biomarkers related to the efficacy of ovarian cancer treatment has also been widely reported. Luo et al. demonstrated that clonal mutations in homologous combination repair pathway were associated with improved survival and chemotherapy response for ovarian cancer (5). Färkkilä et al. reported that tumor mutational signature 3 positivity was associates with prolonged progression-free survival with the combination of niraparib and pembrolizumab (6). Kang et al. proved a 151 DNA repair genes-based score was associated with better survival in ovarian cancer patients treated with platinum-based chemotherapy (7). Based on this evidence, we assume that it is a multi-factor mediated event that influences the therapeutic efficacy of ovarian cancer. Therefore, there is an urgent requirement for a model that could integrates multiple predictive factors for the prognosis and platinum sensitivity in EOC.

This study aims to explore novel predictive genetic characteristics to stratify platinum responsive patients ahead of recurrence. Through deep-sequencing of 60 previously collected EOC tumor samples, we compared the genomic landscape between platinum responsive and resistant patients, encompassing single nucleotide variation, loss of heterozygosity, clonal architecture, and WGD status. Furthermore, the impact of genomic alterations on the response to platinum-based chemotherapy was evaluated. Finally, the lasso regression model integrated with weight coefficient was established for discriminating patients who could benefit from platinum-based therapy, and then TCGA dataset was introduced to validate the reliability of this model.

## Materials and Methods

### Patient Enrollment and Follow-Up

All 60 epithelial ovarian cancer patients treated with standard initial cytoreductive surgery followed by platinum-based chemotherapy were recruited from September 2014 to October 2019. The median age at diagnosis was 55. Among the patients, 50 of them were high grade serous ovarian cancer (HGSOC), and 10 were others including low grade serous ovarian cancer, clear cell ovarian cancer, and endometrioid ovarian cancer. According to regulations 2018 FIGO stages, there were 15 patients with I–II stages and 45 with III–IV stages in this cohort. In addition, all patients received taxol plus platinum-based chemotherapy, and 11 patients received neoadjuvant chemotherapy. The therapeutic evaluation was performed at the ending of surgery and chemotherapy, 54 patients were complete response, and 6 patients were partial response. The demographic characteristics of enrolled patients were showed in **Supplementary Table S1**. All patients were monitored regularly every 3 months at outpatient clinic. PFS was calculated from the date of last chemotherapy to the date imaging diagnosable recurrence. According to the PFS, 44 patients (PFS > 6 months) and 16 patients (PFS < = 6 months) were defined as platinum-sensitive and platinum-resistant recurrence, respectively. The cohort consisted of patients from Beijing Cancer Hospital and Peking Union Medical College, and written informed consent was obtained from all patients before initiating adjuvant chemotherapy for sample collection, gene sequencing and data publication. The study was approved by the institutional review board of Beijing Cancer Hospital (No.2020KT27), Peking Union Medical College (HS-1437) and conducted in accordance with the Declaration of Helsinki. Briefly, the principles of the ethic standard are including social and clinical value, scientific validity, fair subject selection, favorable risk-benefit ratio, independent review, informed consent, respect for potential and enrolled participants, and others.

### Sample Collection

All patients underwent optimal cytoreduction. Primary tumor tissues together with matched normal tissues were collected and prepared into formalin-fixed paraffin-embedded (FFPE) samples. Genomic DNA extraction was performed with TIANamp Genomic DNA kit (Tiangen Biotech, Beijing, China) following manufacturer’s instruction.

### Library Construction and High-Throughput Sequencing

Genomic DNA prepared *via* the methods stated above were fragmented with an ultra-sonicator UCD-200 (Diagenode, Seraing, Belgium), and subsequently purified and size-selected with magnetic Beads (Beckman, MA, USA). The quality of DNA was determined by Qubit 2.0 Fluorometer with Quanti-IT dsDNA HS Assay Kit (Thermo Fisher Scientific, MA, USA). Library construction was then performed using a custom 53M whole exons capturing probe (IDT, IA, USA). Captured libraries were then pair-end sequenced in 100-bp lengths with Geneplus-2000 sequencing platform (Geneplus, Beijing, China) following the manufacturer’s guidance. Raw data from next-generation sequencing was then filtered to remove low-quality reads and adaptor sequence. Reads were further aligned to the reference human genome (hg19) utilizing BWA aligner (version 0.7.10) for mutation calling. All laboratory procedures are followed the biosecurity guidelines of Geneplus Medical Laboratory.

### Genomic Data Analysis

Single nucleotide variants (SNVs) were called by MuTect (8) (version 1.1.4). For quality control, somatic mutations were identified only when (1) present in <1% of the population in the 1000 Genomes Project (https://www.internationalgenome.org/), the Exome Aggregation Consortium (ExAC), and the Genome Aggregation Database (gnomAD) (https://gnomad.broadinstitute.org); (2) not present in paired germline DNA from normal tissues; (3) detected in at least 3 high-quality reads containing the particular base, where high-quality reads were defined with Phred score > = 30, mapping quality ≥ 30, and without paired-end reads bias. Germline mutations were called by GATK (version 4.0) and in-house script. The mutational signature analysis was performed with unfiltered somatic mutations using R package YAPSA (9) and matched to COSMIC signature database (https://cancer.sanger.ac.uk/cosmic/signatures). Somatic copy number variation (SCNV) was identified by GATK (version 4.0). The focal level of somatic copy number variation was detected by in-house script with bam file. The clonal architectures of somatic mutations and status of whole genome duplication (WGD) were inferred by ABSOLUTE (10) considering with tumor purity and copy number alterations. Events with estimated upper 95% confidential intervals of cancer cell fraction (CCF) of 1 were defined as clonal, whereas the rest were defined as sub-clonal. The 276 DNA damage repair (DDR) related genes list was downloaded from previous report (11). LASSO regression model was trained and tested (patients’ proportion is 2:1) with potential biomarkers which have prognostic value and ROC curve was drawn with R package glmnet (version 4.0.2). The TCGA dataset of ovarian cancer was downloaded from cbioportal (http://cbioportal.org/) and PanCanAtlas (https://gdc.cancer.gov/about-data/publications/pancanatlas). Three hundred eighty-eight patients were derived with status of all three genomic features and platinum sensitivity, then 10% of total patients were randomly selected as the test set, and cross-validated for 10 times.

### Statistics

Two-sided Mann-Whitney and Fisher’s exact tests were performed on Graphpad Prism (version 7.01) or R (version 3.6.1) to generate the P value. Log-rank tests within Kaplan-Meier estimation was introduced to study the predictive impact of biomarkers in estimating the progression free survival (PFS). Multivariate Cox regression was performed by R package survminer (version 0.4.8.999) to test whether each biomarker of interest was an independent predictive marker. For all tests, a P value < 0.05 was considered statistically significant.

## Results

### Mutational Landscape of DNA Damage Repair Related Genes in Epithelial Ovarian Cancer Patients

**Figure 1:**
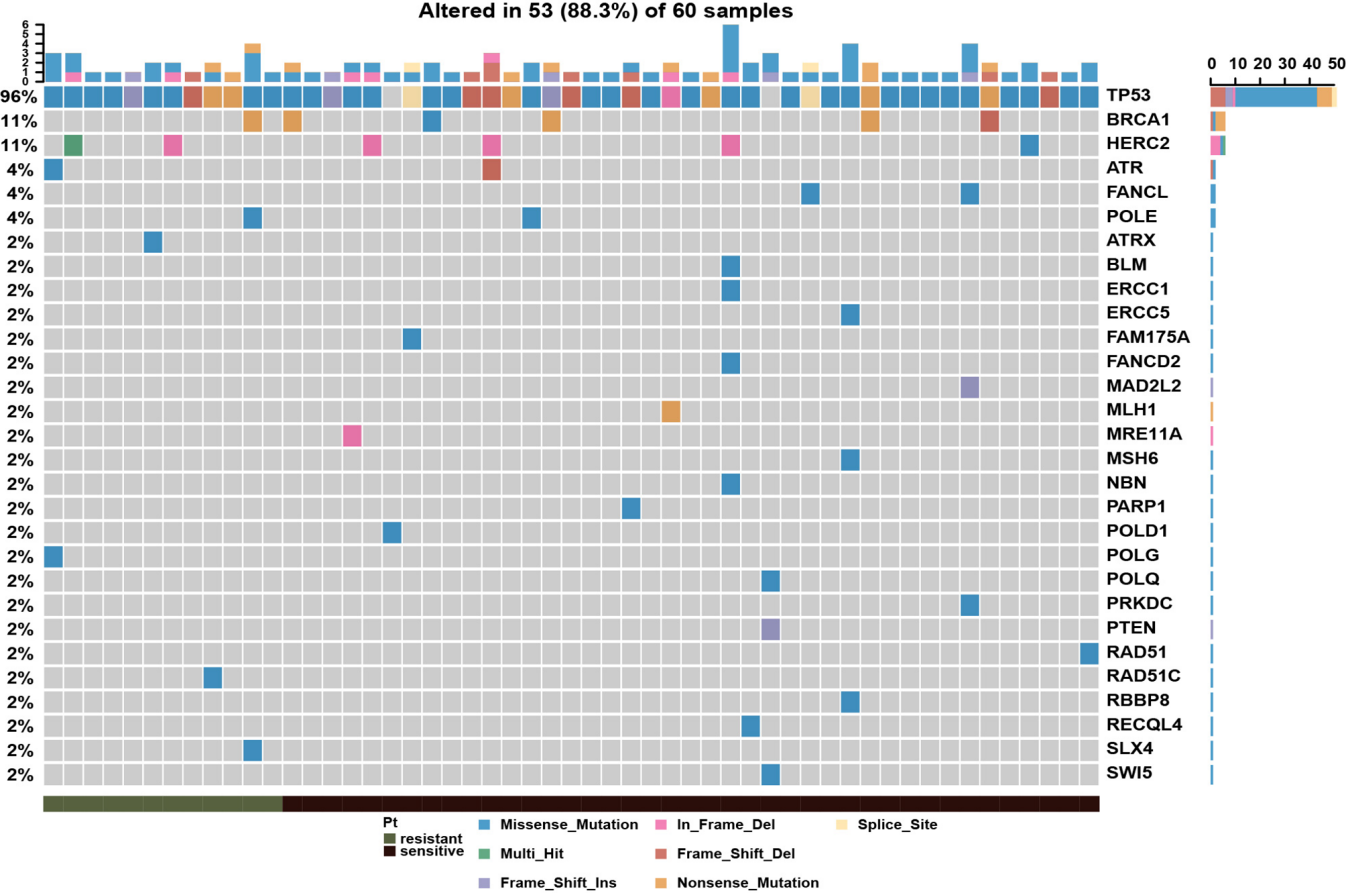
Mutational landscape of DNA damage repair related genes in epithelial ovarian cancer patients, showing with the number of somatic mutations in each patient (top), the mutation frequency of each gene (right), and platinum response status (bottom).

To systematically evaluate the impact of genomic variation on platinum-based chemotherapy, we performed high-throughput sequencing on 60 epithelial ovarian cancer (EOC) patients completed with standard initial treatment. The mean sequencing depth for this cohort was 273X, and the mean mutation count was 134 (**Supplementary Table S2**). According to previous report, the platinum-based chemotherapy could kill tumor cells by binding to DNA and leading to inter-strand or intra-strand crosslinks which would prevent DNA replication (12). The platinum-based treatment efficacy was determined by not only the amount of imposed DNA damage but also by the DNA damage repair (DDR) ability of host individuals. To depict the aberrations on DDR pathway in this cohort, the landscape of diver mutations was showed in **Figure 1**. *TP53* was the top (96%) mutation within 51 patients, who were detected with at least one driver mutations in top 20 frequency. Other DDR drivers recurrently mutated included *BRCA1*, *HERC2*, *ATR*, *FACNL*, *POLE*, *ATRX*, *BLM*, *ERCC1*, *ERCC5*, and others. Meanwhile, the results showed that there was no significant discrepancy on single DDR mutation between platinum-sensitive and platinum-resistant patients. There were 17 patients (28.3%) with germline or somatic *BRCA1/2* mutations. However, as shown in **Supplementary Figure S1A**, *BRCA* mutations were not associated with platinum sensitivity (P = 0.52). Furthermore, patients with *BRCA* mutations had almost overlapped PFS curve compared to patients with wild-type *BRCA* (**Supplementary Figure S1B**, P = 0.78). According to above results, we wonder if there is a comprehensive biomarker could utilize to predict platinum treatment sensitivity instead of just relying on solely single mutation within DDR genes.

### Mutational Signature SBS10a Was Significantly Associated With Better Platinum Response and Prolonged Progression-Free Survival

**Figure 2:**
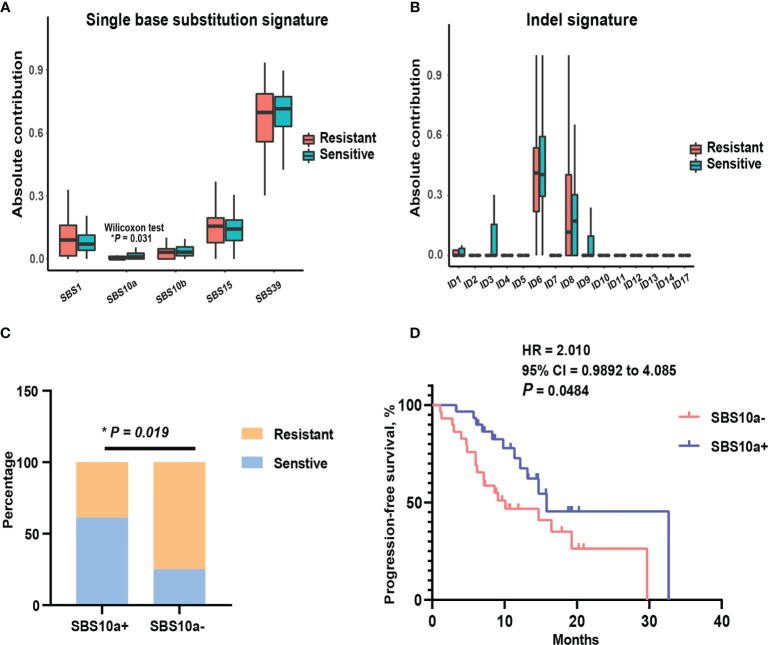
Mutational signature SBS10a was significantly associated with better platinum response and prolonged progression-free survival. **(A)** Comparison of the absolute contribution of each single base substitution signature between platinum sensitive and resistant EOC patients. **(B)** Comparison of the absolute contribution of each indel signature between platinum sensitive and resistant EOC patients. **(C)** The distribution of SBS10a between platinum sensitive and resistant patients. **(D)** The survival curve with log-rank test for SBS10a signature in EOC patients. *P < 0.05.

To assess global characteristics of tumor genome between platinum-sensitive and resistant patients, the mutational signatures of single base substitution, small insertion, and deletion, were compared in these two subsets. Then, the contributions of our cohort were aligned to COSMIC v.3.1 mutation profiles. As shown in **Figures 2A, B**, there was no indel signature with marked difference between two subsets by absolute contributions. Notably, for single base substitution, the absolute contribution of SBS10a was significantly higher (P = 0.031) in platinum-sensitive patients than platinum-resistant patients. SBS10a is predominantly characterized by C > A transversion and is proposed as polymerase epsilon exonuclease domain mutations. Obviously, SBS10a is strongly associated with DDR deficiency and hypermutations. To balance the distribution of patients in SBS10a positive and negative groups, we defined the absolute contribution above 0.5 was SBS10 positive. As a result, 31 patients were classified to SBS10 positive (51.67%) and 29 patients were SBS10 negative (48.33%). Combined with platinum sensitivity information, patients with positive SBS10a were significantly enriched in platinum responder cohort (**Figure 2C**, P = 0.019). Therefore, we wondered if patients with positive SBS10a would have prolonged PFS than SBS10a negative patients. The Kaplan-Meier analysis was utilized to delineate survival curves, as shown in **Figure 2D**; patients with positive SBS10a had significantly improved median PFS of 15.8 months compared with 10.1 months for those with negative SBS10a (95% CI = 0.9892 to 4.085, P = 0.0484, log-rank test). These results indicated that SBS10a positivity was a potential biomarker for stratification of ovarian cancer patients.

### Patients With Loss of Heterozygosity of *FAM175A* Have Potential Clinical Impact

**Figure 3:**
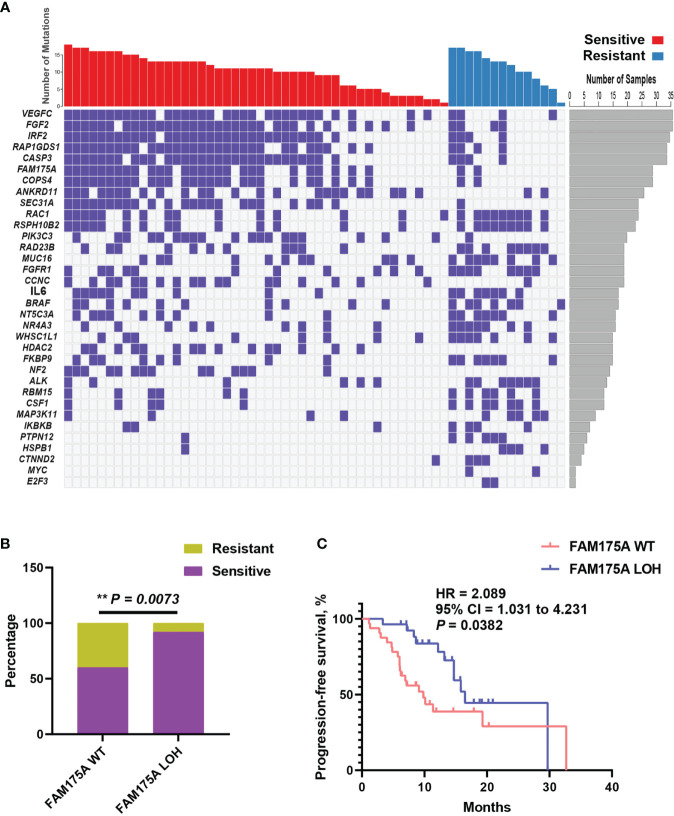
Focal copy number variation analysis identifies FAM175A LOH has potential prognostic value. **(A)** Significantly discrepant focal LOHs with Fisher’s exact test were exhibited between platinum sensitive and resistant patients; left panel, gene symbol; right panel, frequency for each gene. **(B)** The distribution of FAM175A LOH between platinum sensitive and resistant patients. **(C)** The survival curve with log-rank test for FAM175A LOH in EOC patients. **P < 0.01.

The extensive copy number alterations were observed in this EOC cohort. We evaluated copy number variations on gene level across all 60 patients and compared the discrepancy between platinum-sensitive and resistant group. As shown in **Figure 3A**, significantly discrepant focal LOHs with fisher’s exact test were exhibited between two subsets, such as *VEGFC, FGF2, IRF2, RAP1GDS1, CASP3, FAM175A*, and others. Particularly, *FAM175A* encodes a protein that binds to the C-terminal repeats of *BRCA1* and is required for efficient DNA repair of DNA double- strand breaks (13, 14). To evaluate the effect of *FAM175A* on platinum sensitivity and prognostic value, EOC patient enrichment and Kaplan-Meier analysis were performed between *FAM175A* LOH and wild-type group. It is showed in **Figure 3B**, patients with *FAM175A* LOH were significantly enriched in platinum responder cohort (P = 0.0073). Furthermore, patients with *FAM175A* LOH had prominently longer median PFS of 16.5 months than 9.8 months for those wild-type patients (**Figure 3C**, 95% CI = 1.031 to 4.231, P = 0.0382, log-rank test). These results suggested *FAM175A* LOH had potential clinical impact on platinum treated EOC patients. Notably, we also explored the relationship between *FAM175A* LOH and *BRCA* mutation. As a result, *FAM175A* LOH was not associated with *BRCA* mutation (**Supplementary Figure S2A**, P = 0.7721). Therefore, we then asked if *FAM175A* LOH could be a compensation for BRCA mutation as paralleled predictor for platinum efficacy. As shown in **Supplementary Figure S2B–C**, patients with any of *FAM175A* LOH or *BRCA* mutation were significantly enriched in platinum responder cohort (P = 0.0212), and a broadline significant trend of longer PFS than patients without these two variations (95% CI = 0.8303 to 3.949, P = 0.0902, log-rank test). These results suggested that *FAM175* LOH together with *BRCA* mutation could be potential predictor for clinical outcome in EOC patients.

### Whole-Genome Duplication Was Associated With Platinum Resistant and Worse PFS

**Figure 4:**
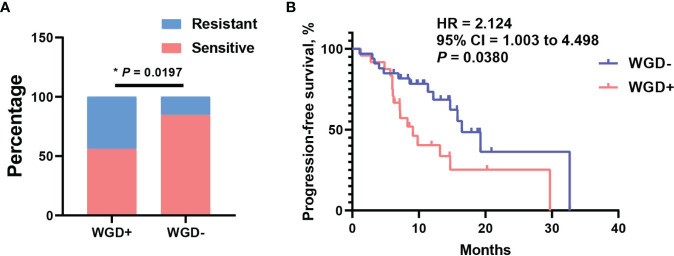
Whole-genome duplication (WGD) was associated with platinum resistant and worse PFS. **(A)** The distribution of WGD between platinum sensitive and resistant patients. **(B)** The survival curve with log-rank test for WGD in EOC patients. *P < 0.05.

The whole-genome duplication was also extensively observed with frequency 43.1% in this cohort. As we known, WGD is a hallmark during tumor evolution, which could shape the clonal architecture and promote tumor progression. To assess what effect exerted by WGD in EOC patients, distribution analysis of WGD patients was performed between platinum-sensitive and resistant subsets. It is shown that patients with WGD were significantly enriched in platinum-resistant cohort (**Figure 4A**, P = 0.0197). Consistently, patients with WGD had significantly worse median PFS of 9.1 months than 16.8 months for those without WGD (**Figure 4B**, 95% CI = 1.003 to 4.498, P = 0.038, log-rank test). Moreover, we wondered that if there was association between WGD and BRCA mutation in this cohort. Distribution enrichment with fisher’s exact test indicated that WGD was not correlated with *BRCA* mutation (**Supplementary Figure S3A**, P = 0.5636). Likewise, to evaluate possibility of WGD and *BRCA* mutation as an integrated predictor for clinical outcome, the association of these two biomarkers with platinum response was analyzed. As shown in **Supplementary Figure S3B**, patients with any of negative WGD or *BRCA* mutation were predominantly enriched in platinum responder cohort (P = 0.0286). Survival analysis revealed that patients with any of these two biomarkers had a favorable trend of better PFS than those presented with positive WGD and wild-type *BRCA* (**Supplementary Figure S3C**, 95% CI = 0.8264–4.193, P = 0.084, log-rank test). These results suggested WGD plus BRCA mutation had potential to predict clinical outcomes in EOC patients.

### Clonal Mutations or Co-Variations in DDR Pathways Have Potential Prognostic Value

To explore other possible biomarkers for clinical outcome of EOC patients with platinum treatment, the intra-tumor heterogeneity (ITH) and co-variations in DDR pathways were investigated. The somatic mutations in DDR pathways were classified into clonal and sub-clonal groups by considering tumor purity, absolute somatic copy number and cancer cell fraction. Unfortunately, clonal DDR mutations were not associated with platinum response (**Supplementary Figure S4A**, P = 0.4858). Nevertheless, survival analysis showed that patients with clonal DDR mutations had longer median PFS 29.7 months than 14.7 months for those only comprised by sub-clonal DDR mutations, with a certain trend toward significance (**Supplementary Figure S4B**, 95% CI = 0.9484–4.514, P = 0.091, log-rank test). Patients with co-variations in DDR pathways were confirmed when any of following situations emerged within 80 core DDR genes: 1), germline mutation plus somatic mutations; 2), somatic mutations plus somatic LOH; 3), two somatic mutations. As a result, co-variations in DDR pathways were not related with platinum response in this cohort (**Supplementary Figure S4C**, P = 0.5408). Notably, it is a clear tendency to significance that patients with co-variations in DDR pathways had better median PFS of 29 months than 13.2 months for those without co-variations (**Supplementary Figure S4D**, 95% CI = 0.9364 to 3.94, P = 0.085, log-rank test). To sum up, clonal mutations and co-variations in DDR pathways are potential prognostic biomarker for EOC patients with clear tendency, however, that warrants future prospective investigation.

### Construction and Validation of Multi-Factor Model for Predicting Platinum Sensitivity in EOC Patients

**Figure 5:**
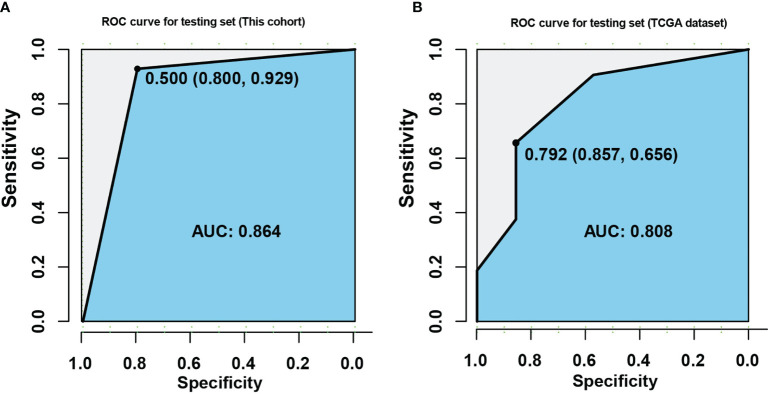
Construction of multi-factor model for predicting platinum sensitivity in EOC patients. **(A)** ROC curve was established with training and testing sets in this cohort. **(B)** ROC curve was established with training and testing sets in TCGA dataset. The X axis indicates specificity, and the Y axis indicates sensitivity.

Five genomic variation events with potential prognostic value were derived from platinum treated EOC patients. Through those candidate events, an integrated model which intended to distinguish platinum beneficial patients, was trained and validated with 60 EOC tumor samples by employing lasso regression. Interestingly, the weight coefficient of clonal mutations and co-variations was compressed to zero by LASSO innate penalty mechanism, due to their less contribution for platinum sensitivity. With SBS10a+, *FAM175A* LOH, and WGD-, 60 patients were divided in training set and testing set. After training, these parameters were fitting with the LASSO regression to generate ROC classification curve. A remarkable performance with the AUC value 0.864 was observed in ROC curve (**Figure 5A**). To validate the reliability of these biomarkers, TCGA dataset of ovarian cancer was downloaded. After training and testing, an AUC value 0.808 was obtained with TCGA dataset, suggesting potential predicting value of these variations in clinical setting (**Figure 5B**). Furthermore, with the purpose of identifying prognostic biomarker, a multivariate Cox regression analysis was performed with those five variations; result showed that wild-type of *FAM175A* was an independent predictor of poor prognosis in platinum treated EOC patients (**Table 1**, HR = 2.759, 95%CI = 1.232–6.175, P = 0.014).

**Table 1:** Multivariate Cox-proportional hazard analyses of the association with PFS using five discrepant variables between platinum sensitive and resistant EOC patients. *P < 0.05.

	Hazard ratios	95% CI of HR	p-value
**SBS10a-**	1.883	0.231–1.218	0.135
***FAM175A* WT**	2.759	1.232–6.175	*0.014
**WGD+**	2.07	0.902–4.464	0.088
**Co-variation-**	1.068	0.264–3.322	0.918
**DDR clonal-**	2.094	0.099–2.301	0.357

The bold value means this variable was significantly associated with PFS in multivariate Cox-proportional hazard analysis.

## Discussion

The overall survival for EOC has not been improved for decades worldwide (15, 16). Since the 1990s, platinum compounds have been the basis of standard of care for ovarian cancer treatment (17–19). Most patients are highly platinum-sensitive at initial treatment with only 20–30% resistant to platinum-based chemotherapy which is defined as PFS less than 6 months (20, 21). PFS is the conventional golden standard for distinguishing platinum-sensitive from platinum-resistant cases in clinic. Thanks to the development of genetic testing technology, we have gotten a better understanding of molecular landscape in ovarian cancer, so that tumor inherent characteristics like platinum sensitivity can be elucidated at initial treatment to assist planning the individualized treatment modality and evaluate the prognosis. Consequently, credible predictive methods to identify platinum sensitivity are warranted.

Ovarian cancer is highly heterogeneous and characterized by a high genomic instability with a high frequency of genomic structural variations such copy number variations (CNVs) and loss of heterozygosity (LOH). *TP53* mutations are presented in most cases, while germline and somatic *BRCA1* or *BRCA2* mutations are presented in around 25% and 8% of cases respectively (22). The high genomic instability is attributable to the universal presence of *TP53* mutations and the frequent alterations in the HR DNA repair pathway (23). The associations between intrinsic genomic features and platinum sensitivity were explored in-depth with NGS technology in several studies (24, 25). However, all those studies derived only single variation, which lacked comprehensiveness and extensiveness. To date, few predictive models encompassing multiple factors has been recognized in capable of identifying response to platinum-based agents. Fekete et al. has compiled an integrated database with 12 repositories from GEO and TCGA to uncover new biomarkers for chemotherapy response in serous ovarian cancer (26). The responder and non-responder cohorts were defined based on duration of relapse-free survival. The results showed that among the top 8 significant genes, *NCOR2, TFE3, AKIP1, PDXK, MARVELD1*, and *AKIRIN2* were validated in an independent cohort to be considered as predictive biomarkers for platinum and taxane resistance. A study published in Nature illustrated the whole-genome characterization of chemo-resistant ovarian cancer (27). The results demonstrated that the inactivation of tumor suppressors *RB1, NF1, RAD51B*, and *PTEN* could contribute to acquired chemotherapy resistance, and *CCNE1* amplification was common in primary resistant cases. In addition, multiple reversions of germline *BRCA1* or *BRCA2* mutations, loss of *BRCA1* promoter methylation, an alteration in molecular subtype, and overexpression of *MDR1* were additional reasons of drug resistance. It was reported that a scoring system for platinum response in high-grade serous ovarian carcinoma was established (28). Gene expression signatures from TCGA-dataset were collected to identify 11 significantly differentially expressed genes associated with platinum response. The results suggested that *HSD11B1* was highly significantly associated with lower risk of recurrence in HGSOC, and response to platinum-based therapy was related to distinct gene-expression patterns of tumor immune-system. Although this scoring system achieved favorable AUC value in predicting platinum sensitivity, the accuracy of the evaluation process is debatable due to the vulnerability of RNA expression, thus DNA based genomic variations are preferable choice for biomarker development.

As discussed above, the development of predictive model for platinum sensitivity all relied on PFS as stratification criteria, so as in our study. We first demonstrated three novel indicators *FAM175A*, SBS10a and WGD using NGS test of tumor samples from 60 patients in predicting platinum sensitivity with favorable application value. Firstly, EOC was predominantly characterized by *FAM175A* LOH in platinum-sensitive patients. *FAM175A* is also known as *ABRAXAS1*, *CCDC98*, or *ABRA1*. Wang et al. concluded that the *RAP80*-*FAM175A* complex may help recruit *BRCA1* to DNA damage sites in part through recognition of ubiquitinated proteins (14). In addition, the low expression of *FAM175A* was demonstrated to have significantly better response to chemotherapy and longer overall survival in non-small cell lung cancer (29). Solyom et al. further proved *FAM175A* c.1082G>A mutation connects to breast cancer predisposition which provided the identity of *FAM175A* as a new breast cancer susceptibility gene shared the mechanism with *BRCA1* (30). The second hallmark of platinum-sensitive EOC patients was mutational signature of SBS10a. As proposed by COSMIC, this signature was probably associated with polymerase epsilon exonuclease domain mutations. Analogously, a recent study reported that tumor mutational signature 3 positivity was associates with *PARP* inhibitor response and prolonged progression-free survival (6). Both of signature 10a and signature 3 are closely related to homologous recombination deficiency, thus we believe SBS10a is a potential efficacy and prognostic indicator. Thirdly, WGD was proved to associate with platinum-resistant in EOC. WGD is a prevalent event during the evolution of cancer genome, involving a complete set of chromosomes doubling (31). Several studies reported that WGD was associated with increasing tumor cell diversity, accelerating of genome evolution and worse prognosis (32, 33). A recent report explored the mechanism of WGD in chemotherapy resistance in colorectal cancer. It was claimed the tumor cells with WGD were less prone to caspase3 activation after chemotherapy treatment (34). In consequence, WGD is a potential biomarker for efficacy evaluation as well.

It is well-known that almost half of high grade serous ovarian cancer is characterized by mutational and functional inactivation of homologous recombination repair, and they are mostly sensitive to *PARP* inhibitors and platinum. HRR mutations, including non-*BRCA* genes, significantly prolong PFS and OS in ovarian carcinoma (35). However, in our cohort, *BRCA* mutations were not associated with platinum sensitivity. So, to eliminate the limitations of a single variation, other HRR related molecular characteristics which could affect or predict platinum sensitivity, were extensively explored. As discussed above, *FAM175A* may help recruit *BRCA1* to DNA damage site and share similar susceptibility with *BRCA1* mutation, thus we assume *FAM175A* may compensate to *BRCA1* in DDR pathways. Through in-depth analysis in our study, it was found that patients with any of *FAM175A* LOH or *BRCA* mutation were significantly enriched in platinum responder cohort, and a significant trend of longer PFS was exhibited than patients without these two variations. It might suggest that *FAM175* LOH could compensate to *BRCA* mutation and could be potential platinum-sensitive predictors in EOC patients. Moreover, Luo et al. reported mutational clonality in DDR pathways may affected chemotherapy sensitivity (5) and Wang et al. revealed co-mutations in DDR pathways could serve as prognostic biomarker (36). Accordingly, these two biomarkers in our cohort were examined and indicated that the mutational clonality or multiple hits of genes involved in DDR pathways had tendency to play a pivotal role in platinum response. Unfortunately, the difference was not statistical significance in this study, but might be significant when expending sample size. Besides, we explored the variation beyond DDR pathway, especially in whole chromosome level. It was demonstrated that WGD was not correlated with *BRCA* mutation, survival analysis revealed that patients with any of non-WGD or *BRCA* mutation had potential to predict better clinical outcomes in EOC patients than those with WGD and wild-type *BRCA*.

Collectively, our data established a promising NGS-based predictive model which was developed by integrating the status of *FAM175A*, SBS10a, and WGD that has been successfully build up to differentiate platinum sensitivity. Besides, *FAM175A* LOH was proved as an independent favourable prognostic factor. To further evaluate clinical performance and promote translational guidance of this model, a prospective study with expanded sample size will be initiated.

## Data Availability Statement

The datasets presented in this study can be found in online repositories. The names of the repository/repositories and accession number(s) can be found below: China National GeneBank DataBase [accession: CNP0001937].

## Ethics Statement

The studies involving human participants were reviewed and approved by Ethics Committee of Beijing Cancer Hospital and Ethic Committee of Peking Union Medical College Hospital. The patients/participants provided their written informed consent to participate in this study.

## Author Contributions

Conceptualization, HZ and TS. Data curation, ZT. Formal analysis, NZ. Funding acquisition, TS. Investigation, HW. Methodology, SZ and CZ. Project administration, XX and YaG. Resources, JY, YY, and JL. Software, MG. Supervision, YX and YuG. Writing—original draft, HZ, TS, SZ, and CZ. Writing—review and editing, YX and YuG. All authors contributed to the article and approved the submitted version.

## Funding

This work was supported by Beijing Natural Science Foundation (7204244).

## Conflict of Interest

CZ, JY, and ZT are current employees of Geneplus-Shenzhen. XX, YY, JL, and YaG are current employees of Geneplus-Beijing.

The remaining authors declare that the research was conducted in the absence of any commercial or financial relationships that could be construed as a potential conflict of interest.

## Publisher’s Note

All claims expressed in this article are solely those of the authors and do not necessarily represent those of their affiliated organizations, or those of the publisher, the editors and the reviewers. Any product that may be evaluated in this article, or claim that may be made by its manufacturer, is not guaranteed or endorsed by the publisher.

## Supplementary Material

Supplementary Figure 1 **(A)** The distribution of BRCA mutation (germline and somatic) between platinum sensitive and resistant patients; **(B)** The survival curve with log-rank test for BRCA mutation (germline and somatic) in EOC patients; * P < 0.05, **P < 0.01, ***P < 0.001.

Supplementary Figure 2 **(A)** The association of BRCA mutation (germline and somatic) with FAM175A LOH; **(B)** The distribution of BRCA mutation (germline and somatic) or FAM175A LOH between platinum sensitive and resistant patients; **(C)** The survival curve with log-rank test for BRCA mutation (germline and somatic) or FAM175A LOH in EOC patients; * P < 0.05, **P < 0.01, ***P < 0.001.

Supplementary Figure 3 **(A)** The association of BRCA mutation (germline and somatic) with WGD; **(B)** The distribution of BRCA mutation (germline and somatic) or WGD between platinum sensitive and resistant patients; **(C)** The survival curve with log-rank test for BRCA mutation (germline and somatic) or WGD in EOC patients; * P < 0.05, **P < 0.01, ***P < 0.001.

Supplementary Figure 4 **(A)** The distribution of clonal mutation (DDR pathway) between platinum sensitive and resistant patients; **(B)** The survival curve with log-rank test for clonal mutation (DDR pathway) in EOC patients; **(C)** The distribution of co-variations (DDR pathway) between platinum sensitive and resistant patients; **(D)** The survival curve with log-rank test for co-variations (DDR pathway) in EOC patients; * P < 0.05, **P < 0.01, ***P < 0.001.
