# Supplementary figures and images for: Construction and Validation of a Platinum Sensitivity Predictive Model With Multiple Genomic Variations for Epithelial Ovarian Cancer
Source: Front Oncol. 2021 Sep 16;11:725264. doi: 10.3389/fonc.2021.725264 (PMC8481766; doi:10.3389/fonc.2021.725264)

# Figure S1

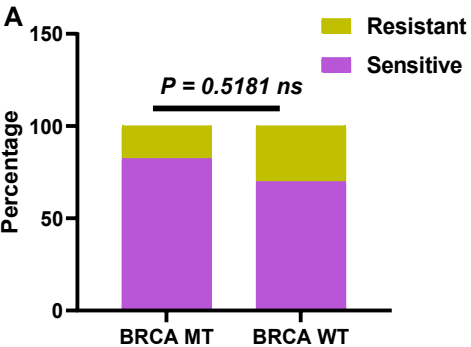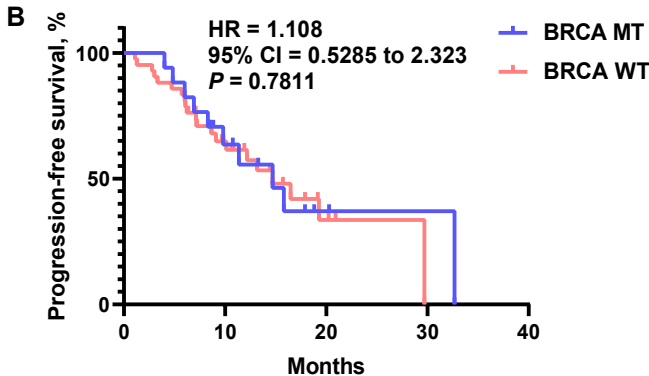

Supplement: Supplementary Figure 1 — (A) The distribution of BRCA mutation (germline and somatic) between platinum sensitive and resistant patients; (B) The survival curve with log-rank test for BRCA mutation (germline and somatic) in EOC patients; * P < 0.05, **P < 0.01, ***P < 0.001. [file DataSheet_1.pdf]

# Figure S2

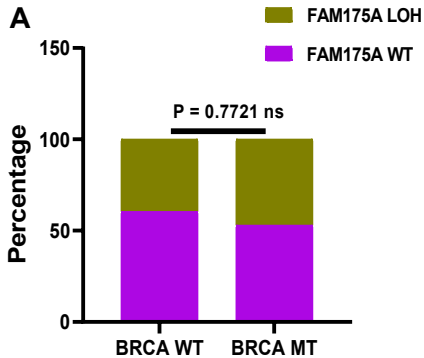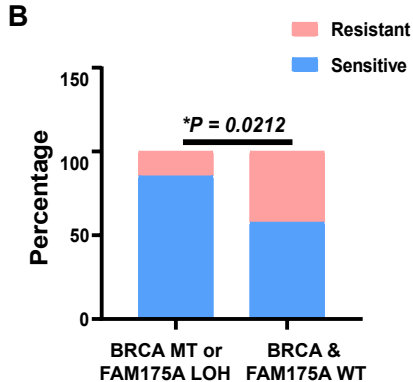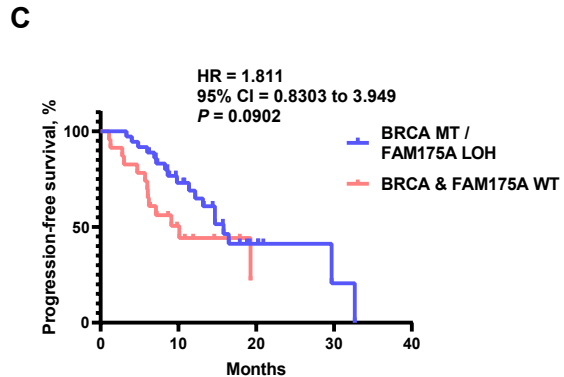

Supplement: Supplementary Figure 2 — (A) The association of BRCA mutation (germline and somatic) with FAM175A LOH; (B) The distribution of BRCA mutation (germline and somatic) or FAM175A LOH between platinum sensitive and resistant patients; (C) The survival curve with log-rank test for BRCA mutation (germline and somatic) or FAM175A LOH in EOC patients; * P < 0.05, **P < 0.01, ***P < 0.001. [file DataSheet_2.pdf]

# Figure S3

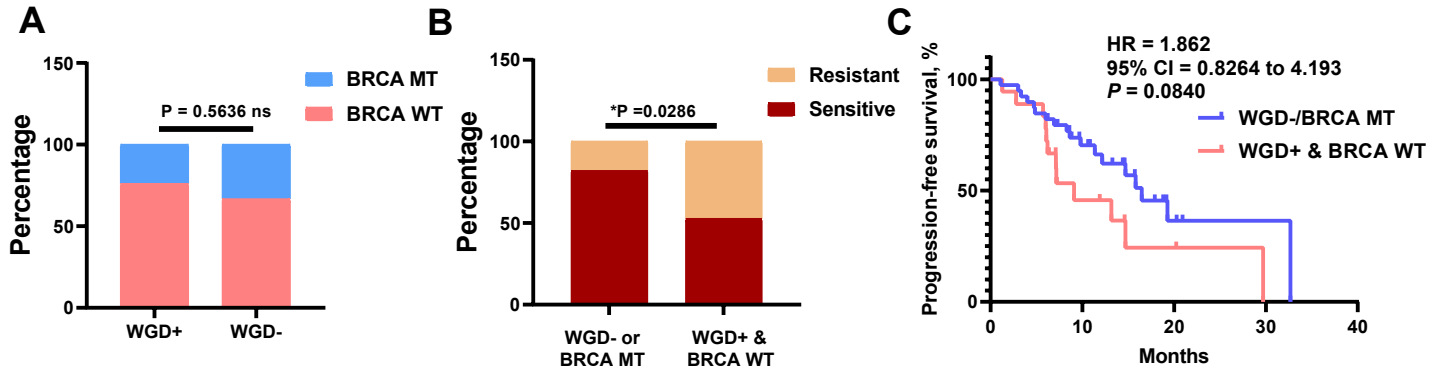

Supplement: Supplementary Figure 3 — (A) The association of BRCA mutation (germline and somatic) with WGD; (B) The distribution of BRCA mutation (germline and somatic) or WGD between platinum sensitive and resistant patients; (C) The survival curve with log-rank test for BRCA mutation (germline and somatic) or WGD in EOC patients; * P < 0.05, **P < 0.01, ***P < 0.001. [file DataSheet_3.pdf]

# Figure S4

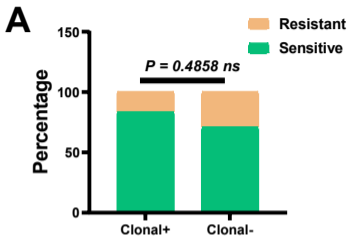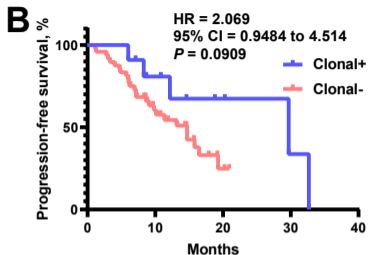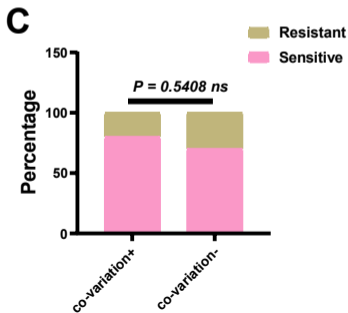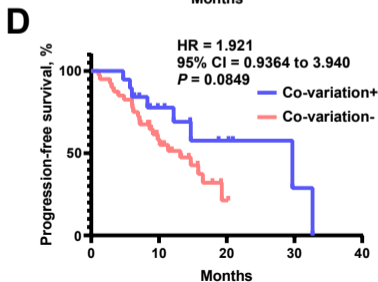

Supplement: Supplementary Figure 4 — (A) The distribution of clonal mutation (DDR pathway) between platinum sensitive and resistant patients; (B) The survival curve with log-rank test for clonal mutation (DDR pathway) in EOC patients; (C) The distribution of co-variations (DDR pathway) between platinum sensitive and resistant patients; (D) The survival curve with log-rank test for co-variations (DDR pathway) in EOC patients; * P < 0.05, **P < 0.01, ***P < 0.001. [file DataSheet_4.pdf]
