# Supplementary material for: Construction and Validation of a Platinum Sensitivity Predictive Model With Multiple Genomic Variations for Epithelial Ovarian Cancer
Source: Front Oncol. 2021 Sep 16;11:725264. doi: 10.3389/fonc.2021.725264 (PMC8481766; doi:10.3389/fonc.2021.725264)
Supplement: Supplementary file 5 [file Table_1.docx]

| Clinical variables | Total number | Platinum-sensitive (n=44) | Platinum-resistant (n=16) | P-value |
| --- | --- | --- | --- | --- |
| Age at diagnosis  > = 60  < 60 | 19  41 | 14  30 | 5  11 | *P* > 0.99 |
| Histology type  HGSOC  Non-HGSOC | 50  10 | 36  8 | 14  2 | *P* = 0.72 |
| 2018 FIGO stage  I-II  III-IV | 15  45 | 14  30 | 1  15 | *P* = 0.049 |
| NACT  Yes  No | 11  49 | 7  37 | 4  12 | *P* = 0.46 |
| Evaluation of initial treatment  CR  PR | 54  6 | 42  2 | 12  4 | *P* = 0.038 |

**Supplementary Table 1. The demographic characteristics of enrolled patients**

HGSOC, high grade serous ovarian cancer; NACT, neoadjuvant chemotherapy; CR, complete response; PR, partial response
